# Experiences of primary eye care use among adults in Southern Ethiopia: A qualitative study

**DOI:** 10.4102/hsag.v29i0.2704

**Published:** 2024-09-06

**Authors:** Temesgen W. Kentayiso, Naomi L. Nkoane, Kholofelo L. Matlhaba

**Affiliations:** 1 Department of Health Studies, College of Human Sciences, University of South Africa, Pretoria, South Africa; 2 Orbis International, Addis Ababa, Ethiopia

**Keywords:** primary eye care, primary eye service use, experience, barriers, primary eye care workers, Southern Ethiopia

## Abstract

**Background:**

Primary eye care (PEC) is an important component of comprehensive eye care services that allows communities to enjoy basic high-quality services. However, because of various determinants, communities do not use this service.

**Aim:**

This study aimed to explore and describe the experience of adults who used PEC services in the last 6 months.

**Setting:**

This study was carried out in four districts in southern Ethiopia from June to September 2023.

**Methods:**

An exploratory descriptive qualitative study design was used to understand the experiences of adults 40 years and older who had used PEC services. Six focus group discussions were conducted with a total of 48 participants. A nonprobability purposive sampling technique was used to draw participants. Data were analysed thematically using ATLAS.ti software version 23.2.2.

**Results:**

Primary eye care service use experiences of adults 40 years and above were discussed in terms of three emerging themes: the experience of community service use, barriers to service and suggestions to improve service. The study identified poor access to services, service provider-related factors and quality and awareness gaps as barriers to the use of PEC services.

**Conclusion:**

Attention to PEC services, integration of eye care with other primary health care services, deployment of service providers, awareness creation and expansion of PEC units are needed to improve PEC service use.

**Contribution:**

The findings will guide community-based intervention plans to reduce avoidable blindness and low vision, thus improving quality of life.

## Introduction

Eye diseases that could be prevented or treated and cause visual impairment and blindness are major health problems in many developing countries. According to the World Health Organisation (WHO 2022a:16) in 2022, around the world, 2.2 billion people were estimated to have visual impairment, either blindness or low vision. Almost half, one billion, have a condition that could have been prevented or treated (WHO 2022b:35). Underdeveloped countries generally carry the greatest burden, with three-quarters of the blind and visually impaired population (Malik, Mafiri & Gilbert 2018:176).

Every year a total of three trillion dollars is spent by the community, family and patient because of visual impairment worldwide, causing a huge economic impact that hinders individual and social development (Forrest et al. 2023:1; Solomon et al. 2022:e116). In addition to the economic and developmental impact, visually impaired people experience hardship and need the help of a family or community member for routine tasks. The hardship will last longer when blindness occurs in childhood (WHO & Unicef 2020:18). Of the regions of the world, the highest prevalence of age-standardised blindness is recorded in sub-Saharan Africa, 4.3% in eastern Africa and 5.1% in western sub-Saharan Africa (Aghaji et al. 2021a:1; Med et al. 2019:3).

Visual impairment and blindness, a problem of more than 15 million Ethiopians, are among the main public health problems in the country. According to the Ethiopian Ministry of Health (2016:16), having a 1.6% prevalence of blindness and a 3.7% prevalence of low vision, Ethiopia is classified as one of those countries with a high prevalence of blindness and low vision (Morka, Yibekal & Tegegne 2020:3). Like many sub-Saharan African countries, in Ethiopia, 91.2% of blindness and 87.4% of visual impairment are avoidable, preventable or treatable (Teshome et al. 2021:3).

Primary eye care (PEC) is one of the 11 components of primary health care and was designed to serve the world’s largest and most marginalised communities. The WHO defined PEC as ‘the most basic eye care available to individuals and families regardless of their socioeconomic status to provide care and identify the disease before it becomes a serious medical problem’ (Gilbert et al. 2021:70). Preventive, Promotive, Curative and Rehabilitative activities accompanied by eye health education, case identification, visual acuity measurement, basic eye examination, diagnosis and timely referral are components of PEC. The main objective of PEC is to contribute to reducing avoidable blindness and low-vision in low and middle-income countries, thus improving quality of life (Kalua et al. 2024:4). Universal eye health coverage underscores the fact that everyone should access quality eye care services nearby without the risk of deprivation (Khanna et al. 2020:335).

There is very little evidence of the need for PEC in Africa. On the continent, only 30% of people have access to haphazardly distributed eye care facilities throughout and within countries (Aghaji 2021b:2; Bright et al. 2018:1). A study conducted in Rwanda to assess the population’s needs for PEC found that the estimated need for PEC and its use in the population was 34% (Bright et al. 2018:1). Similarly, the result of a study conducted in Nigeria to assess barriers to the use of eye care services in rural communities in Edo state showed that the use of eye care in the state was 25.2% (Ebeigbe & Ovenseri 2014:102). A separate study conducted in Rwanda found that a third of the population had the potential to benefit from PEC (Burn et al. 2020:165). In South Africa, PEC units provide a large amount of eye care services to the community with a notable referral link (Lilian et al. 2018:3). Similarly, in Kenya, Gambia, Mali and Zimbabwe, the PEC service is provided as part of the PHC (Aghaji et al. 2020:5). To realise the benefit of PEC, there should be a clear and well-designed communication, referral and feedback mechanism from the household to the tertiary level (Aghaji et al. 2018:4).

The use of an eye care service is defined by WHO as the use of an eye care service by people to prevent and cure eye problems, promote the maintenance of eye health or obtain information about one’s eye health status and prognosis (Morka et al. 2020:4; WHO & UNICEF 2020:10). The definition incorporates components of comprehensive eye care services for the prevention, promotion, treatment and rehabilitation of eye health (Teshome et al. 2021:2). The World Health Organisation identified five elements as the key to measuring acceptance of eye care services: accessibility, availability, accommodation, affordability and acceptability (Graham 2017:86).

Even if the appropriate use of eye care services is highly recommended to reduce the high burden of blindness and low vision, the use of eye care services varies widely throughout the world, ranging from 18% to 82.5% (Morka et al. 2020:2). In developed countries, most of the population uses eye care services. Of the developed states, the use of eye care services was 73.5% in South Korea, 57% in Canada and the United States and 67% in Australia (Park et al. 2017:58). Different factors contribute to this high use of eye care services: the level of awareness, accessibility, availability, affordability and acceptance in this region is high.

A study carried out in Pakistan to measure PEC use found that 45.3% of study participants had used PEC in the past 2 years (Rehman & Sahrif 2021:164). The use of PEC was found to have a significant association with awareness level, age, sex, educational level and a recent visit to an eye care provider (Khanna et al. 2020:335). In many low- and middle-income countries, different barriers to the low use of PEC services were reported: long-term use of traditional remedies, lack of awareness, lack of attention to eye problems, economic restrictions and gender (Bhoosnurmath 2017:14). In the late Ilaka state of Nepal, in addition to poor knowledge of ocular morbidity, the long distance to the units providing services, incomplete service, the absence of the service provider during visits and the lack of skill and knowledge of the service provider were identified as barriers to the use of PEC services (Gnyawali, Bhattarai & Upadhyay 2012:96).

Studies conducted to assess the use of PEC services in different parts of sub-Saharan African countries identified the presence of poor utilisation, which is fenced off by many challenges and barriers. The results of studies carried out at different times and states in Nigeria described that eye care use in the community was 19%, 32% and 38%, the last 38% in Abuja (Aghaji et al. 2018:2; Ebeigbe & Ovenseri 2014:99; Moyegbone et al. 2020:4). Similarly, the rate of use of eye care in the past 2 years in Ghana was 32.2% (Ilechie et al. 2013:8). The result of a study conducted in South Africa to assess the use of eye care patterns found that 73.4% of study participants had not used the eye care service. Age, income, ethnicity and medical conditions such as diabetes and access were identified as barriers to the use of services in the country (Akuffo et al. 2020:10).

Another study conducted in South Africa identified the absence or shortage of trained personnel dedicated to the PEC service, the shortage of medications, unaffordable medications, poor support and supervision, incomplete services and a weak referral link to secondary and tertiary eye care facilities as key challenges in the implementation of PEC in the country (Lilian et al. 2018:13). In Nigeria, the cost of treatment, the distance to the eye care unit, the lack or absence of transportation, the need for an escort, religious beliefs and language were reported as reasons for poor access to PEC services (Ebeigbe & Ovenseri 2014:99). The result of a study conducted in Kenya underscores the barrier to the health system as one of the main causes of poor eye care in the country. The study identified a shortage of trained personnel for eye care units, insufficient skill of the service provider and a service that excludes the poor as barriers related to the system. Distance and sex were also found to affect the use of eye care services in Kenya (Med et al. 2019:2). In summary, in sub-Saharan Africa: Geographic, socioeconomic, and political factors were found determining the use of PEC services. Other factors include lack of infrastructure, corruption, unrest, limited funding for eye care services and the absence or shortage of dedicated eye care personnel (Cicinelli, Marmamula & Khanna 2020:321).

In Ethiopia, the experience of PEC is not well studied. A study conducted in Hawassa, Ethiopia, found that only 23.8% of adults 40 years and older had used PEC services in the last 2 years (Morka et al. 2020:2). Most of the study participants said that eye care units should only be visited during illness, and the study identified a major awareness gap among the study participants (Morka et al. 2020:2). So far, few studies have been conducted to measure the rate of PEC in Ethiopia. This study explored and described the experiences of adults 40 years and above who used PEC services to improve the optimal provision of PEC services that will contribute to the reduction of avoidable blindness and low vision. Reduced blindness and low vision will significantly contribute to improving quality of life.

## Research methods and design

### Study design

An exploratory descriptive qualitative study design was applied to explore and describe the use and barriers of PEC services among adults 40 years and above who utilised PEC services in the four districts of southern Ethiopia. The study design was applied to better understand and describe the experience of the service users.

### Study participants

The inclusion criteria applied were adults 40 years and older who had used PEC services in a public PEC unit in the last 6 months and volunteered to participate in the study. Adults who did not use PEC services, those with comorbidity and those unwilling to participate were excluded from the study.

### Setting

This study was carried out in the districts of South Ari, North Ari, Malle and Bena tsemay in the South Omo Zone, Southern Ethiopia. According to the 2023 annual report of the South Omo Zone Health Department, there is one general hospital, two primary hospitals and 47 primary health care units in the South Omo Zone. Regarding the facilities that provide eye care services, there was one secondary eye care unit located in the general hospital and 41 PECUs in primary health care units.

### Data collection

Data were collected with a pretested Focus Group Discussion (FGD) guide until the saturation level was reached. Data collection was carried out in primary eye care units and selected sites in the village where the community spent time together between June and September 2023. The researcher was the main data collector and ensured the application of all ethical protocols during the study. The data-collection assistant captured the emotions and expressions of the participants to carry out the ideas presented during the discussions.

A nonprobability purposive sampling technique was applied to draw study participants. Deliberately selected 48 participants participated in six FGDs to cover the full range of characteristics of interest. During the current study, eight participants were homogeneously engaged in all of the six FGDs. The main questions of the study were: How do you experience the use of PEC service provision? What are the main barriers to PEC services? What key interventions are needed to improve the service? Probing questions were asked to get adequate information. Focus group discussions were organised and conducted in Amharic, the Ethiopian national language, and the moderators, researcher and research assistant (notetaker), who had a lot of experience in language translation and experience in organised FGD, translated the conversation into English and presented the result.

Written consent was obtained from all study participants prior to the investigation. The average time spent with each FGD was 1 h. The discussion was recorded with an H1 Handy recorder and a Smartphone recorder. The researcher had sufficient time to understand the experiences of the participants during the discussion.

### Data analysis

Data analysis in the qualitative study involves organising, thematising and creating categories and subcategories of data to assist in the interpretation of the study findings (Creswell & Poth 2018). The researcher and the research assistant carefully transcribed the data to ensure the quality of the data. The results of the focus group discussions were thematically analysed in a framework using the electronic-assisted qualitative data analysis method using ATLAS.ti software version 23.2.2 (Ravindran 2019). After data entry, codes, code groups, memos and quotations were created to develop the study. After careful selection and review of the generated codes, the researcher designed three themes, seven categories and 22 codes. To determine the final number of codes, code groups and themes, the researcher applied cooccurrence, density and ground analysis of the codes.

### Trustworthiness

To improve the trustworthiness of this study, the researcher strictly followed the criteria of confirmability, dependability, credibility, authenticity, transferability and minimisation of weaknesses. To increase the confirmability, the researcher adopted the concept of member checking to revert to participants with clarity and confirmation of the narrations shared by the participants, as evidenced by the presentation of direct quotations by the study participants. To ensure dependability, the researcher employed a coder and supervisors to assist with the analysis and interpretation of the findings and finalise the emerging themes. Coding and recoding were performed by the researcher, the supervisor and an independent coder.

To ensure the credibility of the study, the researcher used an H1 audio recorder, a smartphone and hard copies of narratives about the experience of using PEC. The researcher also had a prolonged engagement with the study participants and collected in-depth data that helped the investigator understand the experiences of the participants. The researcher also conducted a peer review and debriefing with the supervisor and independent experts in the PEC to ensure that the research processes were logically conducted and that the results were well reported.

To enhance the authenticity of the study, information was collected directly from adults who used services, and findings were presented with a direct quote from the participant. To ensure the transferability of the study, the researcher selected participants with a wealth of experience and presented the topic under discussion in intensive categories along with the developed themes, categories and subcategories.

### Ethical considerations

This study received ethical clearance from the University of South Africa with reference number 58528660_CREC_CHS_2023. Similarly, the study obtained permission from the South Omo Zone Health Department and the four district health offices. Signed consent was obtained from all study participants prior to the actual investigation. During this study, the researcher strictly followed the national and internationally accepted principles of autonomy, justice, privacy, confidentiality, anonymity and non-maleficence.

## Result

### Demographic data of study participants

During this study, 48 purposively selected adults were involved. All participants were over 40 years old, the majority in the age group of 50–60 years. Thirty-one of the participants were men and nine women. More than half of the participants completed their primary education, Grade 8, according to the Ethiopian Ministry of Education classification. The vast majority were married.

### Themes, categories and subcategories

Data analysis resulted in the development of 3 themes, 7 categories and 22 subcategories. The study result is presented further in the text in themes, categories and subcategories (Table 1). The quote used in this section was presented with a direct link to the transcribed document in ATLAS.ti Version 23.2.2. The first number refers to the quotation line in the document, followed by the number assigned to the participant. The show or scroll button (¶) and the number of the FGD follow the description.

**TABLE 1:** Themes, categories and subcategories of the study.

Themes		Categories	Subcategories
1.	Experience of the use of community service utilisation	1.1. Service-related	1.1.1. Lack of world-class eye care service 1.1.2. Inadequate information 1.1.3. Absence of escort
		1.2. Service provider related	1.2.1. Language barrier 1.2.2. Disrespectful service providers 1.2.3. Seasonal service utilisation 1.2.4. Lack of commitment
		1.3. Service access	1.3.1. Long waiting time 1.3.2. Distance to travel 1.3.3. Financial implications
2.	Barriers to primary eye care services	2.1. Quality gap	2.1.1. Inefficient eye care services 2.1.2. Unspecialised service providers 2.1.3. Service inequity
		2.2. Awareness gap	2.2.1. Lack of information and messaging 2.2.2. Fear of surgery 2.2.3. Use of indigenous knowledge
3.	Suggestions to improve primary eye care services	3.1. Improved service	3.1.1. Service expansion 3.1.2. Attention 3.1.3. Integration 3.1.4. Deployment of service providers
		3.2. Improved utilisation	3.2.1. Accommodative primary eye care units 3.2.2. Awareness creation 3.2.3 Expansion of outreach services

#### Theme 1: Experience of the use of community service utilisation

During the FGD, the study participants shared different experiences, and the researcher classified the experiences into service-related, service provider and service access.

**Category 1.1. Service-related:** The lack of world-class services, inadequate information and lack of escort were presented as service-related factors determining the use of PEC services in the study area. The lack of a world-class eye care service is mentioned as a major factor that hindered the use of the PEC service by many participants. Study participants reported the lack of dedicated rooms to provide service and the lack of basic eye care services during the current study. Sample quote:

‘I came to this unit six months ago and the professionals examined me outdoors and gave me an eye drop, but now I am feeling serious discomfort. I am not happy.’ ([AQ2]6:5, ¶7, FGD Two)

Information inadequacy is another identified service-related subcategory. Many participants mentioned that the information they received about their specific eye condition before, during and after treatment from the service providers was inadequate. During the current study, the presence of people with eye complaints who did not receive service because of lack of adequate information was reported. As part of the information shortage, some of the study participants mentioned that the community considers the information they receive from someone they know about eye care services to be more than they do from PEC workers. Sample quote:

‘I came to this PECU after seeing the positive result of the surgery in my cousin. Similarly, others stayed home looking at failed surgery or treatment. Therefore, the service provider must be concise and provide adequate information. We trust the word of someone we know more than the service providers.’ (5:13, ¶15, FGD Three)

**Category 1.2. Service provider related:** Language barriers, disrespectful service providers, seasonal use of services and lack of commitment were presented as determinants related to service providers for the use of PEC services. In the current study, the difficulties of communicating with service providers were presented as a challenge. A participant in the focus group discussion said:

‘There are so many determinants of the use of primary eye care services in the nearby clinic. If you come to this unit after going through a transportation problem, there is a language barrier with the service providers. It is very difficult to communicate easily.’ (6:19, ¶18, FGD Two)

The presence of disrespectful service providers is the second subcategory. The participants explained that respect and a welcoming environment are as important as quality treatment. A participant said that:

‘There exists a misconduct among service providers. They must be polite and caring. Nurses ignore us, we do not go to the primary eye care unit for recreation, we are in pain, so we need proper treatment.‘ (5:15, ¶18, FGD Three)

Participants also complained that service providers spent most of their time doing nonwork-related tasks, such as playing video games, rather than providing basic eye health services. Most of the study participants believe that service providers must be ethical and caring to improve the use of PEC services.

Even if eye care services should be available and used all the time, because of geography and community preferences, use in the four districts of the southern Omo zone in Ethiopia was reported to be seasonal. This use of seasonal services was testified by many participants in the study. Sample quote:

‘Most partially or blind people are poor. They prefer the free service and wait at home until the season of the free outreach programme arrives. They are not coming here fearing the cost of treatment and prefer to stay home for the free service.’ (1:7, ¶12, FGD Five)

Similarly, the lack of commitment of some service providers was reported to be the instigator of the low use of PEC services in the study area. Participants in the focused group discussion listed commitment as a mandatory expertise that an eye care service provider should possess:

‘When I came to this site four months ago with an eye full of discharges, I noticed the presence of a serious gap among service providers. They let me wait a long time for no reason while playing a mobile game.’ (1:5, ¶10, FGD Five)

**Category 1.3. Service access:** Long waiting times, distance to travel and financial implications were access-related determinants identified during the current study. The study mentioned waiting longer than the average time to get the service, which causes discomfort and lack of interest in coming back for more services. A participant said:

‘I had to wait more than four hours to get service after coming to this unit and travelling more than an hour and a half. It was not fair to wait that long to get service.’ (5:1, ¶3, FGD Three)

The participants attributed the long waiting time to the shortage of trained personnel and the incapacity of the PEC unit.

Universal eye health coverage underscores that everyone should access quality eye care services without the risk of deprivation. However, many participants in this study mentioned that distance is their main concern when thinking about the use of PEC services. Sample quote:

‘The distance to this primary eye care unit is the main barrier to accessing eye care services. For example, I travelled more than two hours to get here. Imagine how an old blind guy comes here alone!.’ (5:8, ¶11, FGD Three)

The direct and indirect cost of treatment was also reported as determinants of access to the PEC service in the current study. A study participant mentioned that:

‘Cost is another issue. As you know, we are all poor and concerned that if we pay 100 Ethiopian birr for transportation to the eye care service, where do we get money to feed our children?.’ (2:16, ¶16, FGD Four)

#### Theme 2: Barriers to primary eye care services

In this study, quality and awareness gaps were presented as a category of barriers to PEC services.

**Category 2.1. Quality gap:** During the current study, the presence of inefficient eye care services, unspecialised service providers and service inequity was identified as quality gap-related barriers to the use of PEC services. Many participants in the study describe the efficiency of eye care services as an important indicator of the ultimate use of services. A participant in the focus group discussion reported on the inefficiency of the eye care service:

‘I prefer not to return to this unit for eye care after seeing the service provided last time. It was not well-organised. We want complete and reliable eye care services. I think that is our right.’ (6:6, ¶8, FGD Two)

Service providers are at the heart of PEC service provision. Most of the study participants described the quality and capacity of the service provider as a primary indicator of the decision to use eye care services. Equity in eye care services is presented as one of the key building blocks of eye care services by the WHO (Graham 2017:86). Participants in the focused group discussion mentioned that they are not allowed to freely enjoy this human right.

A participant denounced the quality of care in nearby PEC units by comparing the service he received with the standard of the national average eye care service:

‘We are left-over citizens of this country. This is a country where there is corneal transplant; I remember the former president of this country, Girma Wolde Giorgis, who donated his cornea when he died. However, eye care services lack adequate attention when you go to the community, the lower structure. Look what we are getting here. Counseling and eye drops only. This clearly shows the quality of service.’ (6:18, ¶16, FGD Two)

**Category 2.2. Awareness gap:** In this study, lack of information and messaging, fear of surgery and use of indigenous knowledge were presented as subcategories of awareness gaps. The study participant revealed the lack of information and message in the study area by identifying the community’s consideration of blindness as a fragment of the natural ageing process or as a result of poor behaviour rather than a disease. Saying:

‘Almost everyone in my village thinks blindness is part of the natural ageing process and thinks that there is no treatment for it.’ (3:13, ¶18, FGD One)

‘Some people also consider blindness to be a result of bad doing.’ (5:9, ¶12, FGD Three)

Many study participants present fear of surgery as a barrier to the use of services:

‘There is a fear of surgery in the community. Most people with eye complaints believe that surgery will cause permanent loss of vision rather than correction of vision.’ (1:11, ¶15, FGD Five)

The desire to use and rely on indigenous medications was the last subcategory of the awareness gap identified during the current study. The participants mentioned that many residents of the study area prefer to use indigenous knowledge and remedies for their ocular complaints:

‘There have been traditional remedies for eye problems in this community for a long time. Like epilation (plugging of the inverted eyelashes) and removal of pterygium from the cornea. The community considers this as a single treatment for their problem.’ (4:9, ¶14, FGD Six)

#### Theme 3: Suggestions to improve primary eye care services

To improve the use of PEC services in the study area, participants suggested six areas of improvement presented in two categories of improvements in service and use.

**Category 3.1. Improved service:** The expansion, attention, integration, and deployment of more service providers were suggested to improve the use of PEC services in the study area.

Participants in the focus group discussion feel that the PEC service is not accessible enough and that the expansion of the service is mandatory for better use. Most of the study participants recommended expanding and integrating the service into the rural community to improve its use:

‘If the government made the service available nearby, no one would think twice to get the service. There will be distance, cost or fear of having someone to accompany them when considering using primary eye care services, especially among the elderly, blind, and those with serious eye conditions. I am sure everyone will come and enjoy the service and our village will be free of eye problems.’ (3:24, ¶26, FGD One)

Many participants noted that eye care services did not receive adequate attention in the study area and suggested the integration of the activity with other tasks in the primary care unit:

‘The primary eye care unit shall integrate eye health care services with other outreach activities. Nurses come to our village every month and provide services to children and pregnant mothers. It would be nice if eye care activities were integrated with these activities.’ (5:22, ¶23, FGD Three)

Many study participants pointed to the shortage of service providers in the nearby PEC unit as a barrier to service usage and suggested the deployment of more service providers for better coverage.

**Category 3.2. Improved utilisation:** To improve the use of PEC services, participants in the discussion proposed accommodating PEC services, raising awareness and expanding outreach. Primary eye care units should be suitable for patients with visual impairment. The study participants suggested that standardisation of the PEC unit is necessary to expand the service and increase its use. One of the participants said:

‘The service provision unit shall be differentiated as a unit is expected to provide service for fully and partially blind patients.’ (5:20, ¶22, FGD Three)

The lack of adequate awareness was discussed as a barrier to PEC services in Theme 2. To reverse the condition, participants suggested creating awareness. One participant mentioned the advantage of awareness with the benefits she received saying:

‘Awareness gap is the main barrier. If the community knows better, transportation and economic issues will not be a concern. To your surprise, I walked 26 km to get eye care service before the establishment of this primary eye care unit when my graduate child told me that my eye condition would improve if I received treatment.’ (2:14, ¶15, FGD Four)

Finally, the study participants suggested the organisation of integrated outreach service provision to improve the use of PEC services:

‘I strongly recommend expanding eye care services to villages and organising more outreach programmes. If the service is close, no one will stay home.’ (3:28, ¶28, FGD One)

The result of the study presented earlier in the text was generated from the six FGDs. The researcher created codes for the study to reflect the perception of the community. Supplementary Figure 1 displays the distribution of the code document in a Sankey diagram.

## Discussion

### Theme 1: Experience of the use of community service utilisation

This study explored and described the experiences of adults 40 years and above who utilised PEC services. The lack of world-class services, inadequate information and lack of accompanying persons have been reported as factors affecting the provision of services. Participants reported a lack of world-class services in the field, referring to the treatment they received. This finding is consistent with those of previous studies conducted in Ethiopia and Nepal (Gnyawali et al. 2012:97; Teshome et al. 2021:7). Most participants believed that service providers should be ethical and caring to improve the use of PEC services in the study area. Unfortunately, some service providers were labelled unethical and disrespectful by the study participants (Khanna et al. 2020:338).

The inadequacy of information regarding their particular eye condition was also a service-related factor behind their low use. The participants emphasised that accurate and appropriate information is necessary to make well-understood decisions and to cognise the possible consequences of treatment. This finding is consistent with the results of a study conducted in Edo state of Nigeria (Ebeigbe & Overseri 2014:101).

Language barriers, disrespectful service providers, seasonal use of services and lack of commitment were determinants observed related to PEC during the current study. The participants repeatedly mentioned the presence of language barriers, and the finding is consistent with the results in different sub-Saharan African regions (Cicinelli et al. 2020:321; Ebeigbe & Ovenseri 2014:99). Season-based service use and provision and the lack of commitment of service providers were also reported as service provider-related determinants of PEC service use (Gnyawali et al. 2012:97).

Universal Eye Health Coverage strongly advises that eye health services must be equitable, accessible, comprehensive, high-quality and affordable for all (WHO 2022c:16). The current study revealed that long waiting times, distance to travel and financial implications were access-related determinants of low use of PEC services. Longer than average waiting times have been reported to affect the interest of the community in seeking services (Cicinelli et al. 2020:321).

Many participants in the study reported the distance from PEC units as a reason for the low use of PEC services in the four districts of the southern Omo area of Ethiopia. In addition to distance, the cost of transportation is also identified as a determinant of the use of PEC services. This result is consistent with those of previous studies (Khanna et al. 2020:335; Gnyawali 2012:96). The financial implications are the last determinant of the use of PEC services in the study area. Direct and indirect treatment costs were also found to determine the use of the PEC service (Graham 2017:86; Khanna et al. 2020:335).

### Theme 2: Barriers to primary eye care services

The WHO identified five key elements to measure acceptance of eye care services: accessibility, availability, accommodation, affordability and acceptability (Graham 2017:86). The current study revealed the presence of a gap between these five key elements. Quality and awareness were identified as barriers to PEC services. The quality gap incorporates the inefficiency of eye care services, the presence of unspecialised service providers and service inequity. The participants strongly indicated the need to provide efficient eye care services for better coverage. The preference for specialised service providers has been reported to affect the provision and use of PEC services in the study area (Aghaji et al. 2018: 4). During the current study, the inequity was identified as the poor quality of the service provided and the lack of basic medications and supplies for eye care services. This finding is consistent with the results of a study conducted in South Africa (Cicinelli et al. 2020:321; Lilian et al. 2018:13).

The lack of awareness is one of the barriers to the use of PEC services identified in the current study. This is evidenced by the lack of information and messages, fear of surgery and the use of indigenous remedies for ocular complaints. Many participants saw blindness as part of the natural ageing process and did not have a remedy (Morka et al. 2020:2). Fear of surgery has also been identified as a barrier to service. This result is consistent with the results obtained in Nigeria (Ebeigbe & Ovenseri 2014:100). The study also identified the use of indigenous knowledge to treat eye conditions, including the removal of inturned eyelashes and pterygium, which significantly complicates the treatment results (Aghaji et al. 2020:6).

### Theme 3: Suggestions to improve primary eye care services

To improve the use of PEC services, study participants suggested improving the delivery of services. Primary eye care service expansion, attention to PEC services and deployment of trained eye care professionals have been suggested to improve this service. Most of the study participants recommended expanding and integrating the service into the rural community for better use. This recommendation aligns with the WHO’s recommendation to improve PEC service provision and utilisation in the sub-Saharan Africa region (Graham 2017:85; WHO 2022a:16). Provision of adequate attention was suggested to solve the lack of roads and a scarcity of clean water. The result aligns with a study result in Nigeria (Aghaji et al. 2018:3).

Similarly, awareness creation, accommodative PEC units and expansion of outreach services have been suggested to improve the use of services. During the current study, similar to the result of a study conducted in rural Nigeria, participants suggested awareness creation to solve the poor use of PEC services in the study area (Ebeigbe & Ovenseri 2014:100).

### Implications of the study

This study identified and presented the experience of adults who used PEC services in the last 6 months. Therefore, the results will help policymakers, decision makers and implementers design a community-based intervention plan to improve the optimal provision of PEC services that contribute to reducing avoidable blindness and low vision.

### Limitations of the study

This study explored and described the use of PEC services and barriers from adult service users only. Moreover, the study had a small sample size and cannot be generalised to other settings. Therefore, more studies with service providers and facilities would be appropriate.

## Conclusion

Understanding the experiences of service users is vital for improving service provision. This study identified key determinants of PEC service use in adults who had used the service in the last 6 months. The study revealed gaps in accessing, accepting and affording PEC services, which are key elements in measuring the use of PEC services. The barriers identified in this study need attention and further studies are needed to improve the optimal provision and use of services. Optimal use of PEC services will contribute to reducing avoidable blindness and low vision, which in turn improves quality of life.
